# Correlation analysis between 2D and quasi-3D gamma evaluations for both intensity-modulated radiation therapy and volumetric modulated arc therapy

**DOI:** 10.18632/oncotarget.12279

**Published:** 2016-09-27

**Authors:** Jung-in Kim, Chang Heon Choi, Hong-Gyun Wu, Jin Ho Kim, Kyubo Kim, Jong Min Park

**Affiliations:** ^1^ Department of Radiation Oncology, Seoul National University Hospital, Seoul, Republic of Korea; ^2^ Institute of Radiation Medicine, Seoul National University Medical Research Center, Seoul, Republic of Korea; ^3^ Biomedical Research Institute, Seoul National University College of Medicine, Seoul, Republic of Korea; ^4^ Center for Convergence Research on Robotics, Advanced Institutes of Convergence Technology, Suwon, Republic of Korea; ^5^ Department of Radiation Oncology, Seoul National University College of Medicine, Seoul, Republic of Korea; ^6^ Department of Radiation Oncology, Ewha Womans University School of Medicine, Seoul, Republic of Korea

**Keywords:** 2D gamma evaluation, quasi-3D gamma evaluation, pre-treatment patient-specific quality assurance, intensity-modulated radiation therapy, volumetric modulated arc therapy

## Abstract

The aim of this work was to investigate correlations between 2D and quasi-3D gamma passing rates. A total of 20 patients (10 prostate cases and 10 head and neck cases, H&N) were retrospectively selected. For each patient, both intensity-modulated radiation therapy (IMRT) and volumetric modulated arc therapy (VMAT) plans were generated. For each plan, 2D gamma evaluation with radiochromic films and quasi-3D gamma evaluation with fluence measurements were performed with both 2%/2 mm and 3%/3 mm criteria. Gamma passing rates were grouped together according to delivery techniques and treatment sites. Statistical analyses were performed to examine the correlation between 2D and quasi-3D gamma evaluations. Statistically significant difference was observed between delivery techniques only in the quasi-3D gamma passing rates with 2%/2 mm. Statistically significant differences were observed between treatment sites in the 2D gamma passing rates (differences of less than 8%). No statistically significant correlations were observed between 2D and quasi-3D gamma passing rates except the VMAT group and the group including both IMRT and VMAT with 3%/3 mm (*r* = 0.564 with *p* = 0.012 for theVMAT group and *r* = 0.372 with *p* = 0.020 for the group including both IMRT and VMAT), however, those were not strong. No strong correlations were observed between 2D and quasi-3D gamma evaluations.

## INTRODUCTION

Both intensity-modulated radiation therapy (IMRT) and volumetric modulated arc therapy (VMAT) techniques provide excellent dose conformity to the target volume while minimizing the dose to normal tissue [1]. The IMRT technique achieves optimal dose distributions by modulating multi-leaf collimator (MLC) positions while VMAT generates optimal dose distributions by modulating MLC positions, gantry rotation speeds and dose rates, simultaneously [2]. These modulated delivery techniques can involve large uncertainties in the treatment planning process as well as beam delivery [3–5]. Therefore, patient-specific quality assurance (QA) before treatment has been strongly recommended for both IMRT and VMAT to verify plan delivery accuracy [6–10].

As a verification method for IMRT plans, 2D gamma evaluation by measuring a planar dose distribution has been widely adopted in the clinic [11]. However, recent studies by Stasi *et al*. and Nelms *et al*. demonstrated that no correlation was observed between the results of 2D gamma evaluation and clinically relevant patient dose errors for IMRT [12, 13]. For VMAT, Mancuso *et al*. demonstrated that there were no statistically significant differences in the 2D gamma passing rates between IMRT and VMAT [8]. Betzel *et al*. showed that VMAT deliveries were more tolerant to variations in gantry positions and MLC leaf positions than IMRT deliveries [14]. Heilemann *et al*. and Fredh *et al*. recommended stricter 2D gamma criterion should be used for pre-treatment QA of VMAT plans than IMRT QA [15, 16]. They recommended to use a gamma criterion of 2%/2 mm for VMAT plans rather than 3%/3 mm which is widely used for 2D gamma evaluation of IMRT plans in the clinic. Kim *et al*. recommended to use a 2D gamma criterion of 2%/1 mm for stereotactic ablative radiotherapy (SABR) with VMAT techniques using fine resolution MLCs such as high-definition MLC^TM^ (HD-MLC^TM^, Varian Medical Systems, Palo Alto, CA, USA) [17]. The consensus on pre-treatment QA for VMAT and IMRT still seems to be ambiguous and disputable.

Besides 2D gamma evaluation, various verification methodologies for IMRT and VMAT have been suggested. Several groups suggested to analyze log files registered by the linac control system during beam delivery for the verification of IMRT or VMAT deliveries [18, 19]. However, this methodology has inherent limitations because the verification system and the beam delivery system are not independent of each other. The other approach is to calculate modulation indices [4, 5, 20–24]. The modulation index has limitations since it is based on the calculation with parameters acquired from treatment plans not the measurement. In other words, delivery accuracy can be predicted with the modulation index, however, we cannot verify actual delivery accuracy during beam delivery with the modulation index. For example, the modulation index cannot detect errors such as a network error or machine malfunction. On the other hand, 3D or quasi-3D gamma evaluations can be performed with recently introduced commercial dosimeters or 3D gel dosimeters. Gel dosimeters can acquire 3D dose distributions directly, however, measuring accuracy is not high enough to be used in the clinic yet [25, 26]. Nakaguchi *et al*. validated the COMPASS^TM^ system (IBA Dosimetry, GmbH, Germany) for pre-treatment patient-specific IMRT QA, which is a quasi-3D dose verification system. Sdrolia *et al*. reported institutional tolerances for prostate VMAT QA using the COMPASS^TM^ system [27]. Gueorguiev *et al*. compared the sensitivity of the results acquired using the COMPASS^TM^ system to the point dose measurements and 2D gamma evaluations for IMRT [28].

The previous studies validated the quasi-3D verification method for IMRT and VMAT, however, no study has been performed to investigate the correlation between the results of 2D and quasi-3D gamma methods based on patient CT images for both IMRT and VMAT [29–31]. Rajasekaran *et al*. performed correlation analysis between 2D and 3D gamma evaluation metrics for VMAT. However, the analysis was not performed based on the patient CT images since they used the OCTAVIUS 4D^TM^ system (PTW, Freiburg, Germany) [32]. Similarly, Jin *et al*. also performed correlation analysis between 2D and quasi-3D gamma evaluations, however, it was performed only for VMAT plans with the ArcCHECK^TM^ and 3DVH^TM^ software (Sun Nuclear Corporation, Melbourne, FL, USA) [33]. Therefore, we performed a comprehensive correlation analysis between 2D and quasi-3D gamma evaluations for pretreatment patient-specific QA for both IMRT and VMAT plans using the COMPASS^TM^ system in this study. In addition, we investigated the tendency of 2D and quasi-3D gamma evaluations according to the modulation degree of treatment plans as well as the delivery technique.

## RESULTS

### Log file analysis

The deviation of the MLC leaf position was less than 2.5 mm for all delivered fields including both IMRT and VMAT. The mean and maximum root mean sqruare (RMS) values of MLC leaf motion errors were 0.5 mm and 0.8 mm, respectively, for both IMRT and VMAT. In the case of VMAT, the largest MU deviation was observed at the starting control point of each arc for VMAT delivery, which ranged from -0.06 MU to 0.08 MU. The largest gantry angle deviation was also observed at the starting control point, ranging from -0.8° to 0.9°. Between the original dose distributions of a treatment plan and the reconstructed dose distributions with log files, no clinically significant differences were observed.

### 2D gamma evaluation

**Figure 1:**
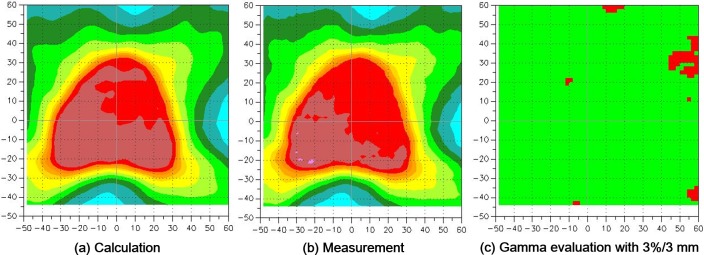
A prostate volumetric modulated arc therapy (VMAT) example of 2D gamma evaluation with a gamma criterion of 3%/3 mm using EBT2 films is shown **a**. the calculated dose distribution with a treatment planning system. **b**. measured dose distribution with EBT2 films. **c**. gamma map. In the gamma map, the passed and failed points are shown in green and red, respectively.

Data for 2D gamma evaluations with EBT2 films are shown in Table 1. An example of 2D gamma evaluation of prostate VMAT with 3%/3 mm is shown in Figure 1. The prostate group with both 2%/2 mm and 3%/3 mm and the VMAT group with 2%/2 mm followed the normal distribution of the Shapiro-Wilk test (*p* > 0.05).

**Table 1:** Summary of 2D gamma evaluation with EBT2 films as well as quasi-3D gamma evaluation with the COMPASSTM system

Group	*N*	Passing rate (%)	*p* value of Shapiro-Wilk test	*p* value of statistical significance of difference (Wilcoxon test)	Confidence limit (%)
**2D Gamma evaluation with 2%/2 mm**					
IMRT	20	86.4 ± 8.3	0.013	0.658	69.1
VMAT	20	84.6 ± 9.2	0.084		66.5
					
Prostate	20	89.1 ± 5.5	0.775	*0.030*	78.2
H&N	20	81.9 ± 10.0	0.039		61.0
					
Total	40	85.6 ± 8.7	0.002		68.0
**2D Gamma evaluation with 3%/3 mm**					
IMRT	20	94.5 ± 5.2	< 0.001	0.469	83.7
VMAT	20	93.0 ± 6.5	0.002		79.4
					
Prostate	20	96.5 ± 2.1	0.200	*0.008*	92.3
H&N	20	90.9 ± 7.2	0.002		75.9
					
Total	40	93.8 ± 5.9	< 0.001		81.9
**3D Gamma evaluation with 2%/2 mm**					
IMRT	20	98.6 ± 0.7	0.011	*0.006*	97.1
VMAT	20	96.6 ± 4.1	< 0.001		88.0
					
Prostate	20	98.5 ± 0.4	0.276	0.295	97.7
H&N	20	96.7 ± 4.2	< 0.001		87.9
					
Total	40	97.6 ± 3.0	< 0.001		91.5
**3D Gamma evaluation with 3%/3 mm**					
IMRT	20	99.6 ± 0.3	0.091	0.191	99.0
VMAT	20	99.0 ± 1.1	0.005		96.7
					
Prostate	20	99.6 ± 0.3	0.128	0.127	99.0
H&N	20	99.0 ± 1.1	0.003		96.7
					
Total	40	99.3 ± 0.9	< 0.001		97.5

*Abbreviations*: *N*, the number of analysis plans; IMRT, intensity-modulated radiation therapy; VMAT, volumetric modulated arc therapy; H&N, head and neck

For the delivery technique, no differences in gamma passing rates with both 2%/2 mm and 3%/3 mm were observed between IMRT and VMAT (all with *p* > 0.05). For the treatment site, the averaged gamma passing rate of the prostate group with both 2%/2 mm and 3%/3 mm were considerably better than those of the H&N group with statistical significance (89.1% ± 5.5% *vs*. 81.9% ± 10.0% with *p* = 0.03 for 2%/2 mm and 96.5% ± 2.1% *vs*. 90.9% ± 7.2% with *p* = 0.008 for 3%/3 mm). This was due to the high modulation of H&N plans than that of prostate plans, which was the same result as the previous study [20].

The corresponding confidence limits (CLs) for each analysis were calculated with proper confidence coefficient respectively. As shown in the Table 1, institutional tolerance levels for IMRT and prostate plans were higher than those for VMAT and H&N plans, respectively, since the average gamma passing rate of IMRT and prostate plans were higher than those of VMAT and H&N plans.

### Quasi-3D gamma evaluation

**Figure 2:**
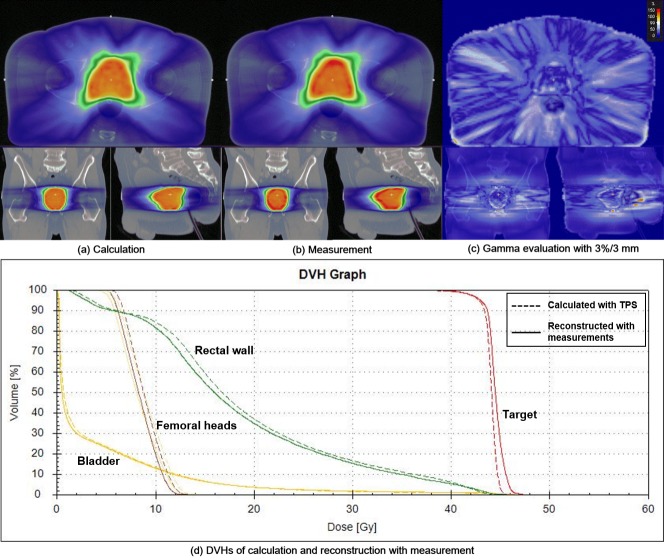
A prostate volumetric modulated arc therapy (VMAT) example of quasi-3D gamma evaluation with a gamma criterion of 3%/3 mm using the COMPASS system is shown **a**. the calculated dose distribution with a treatment planning system (TPS). **b**. reconstructed dose distribution with measured fluences. **c**. gamma map. **d**. dose volume histograms (DVHs). The DVHs of the target volume, rectal wall, bladder and femoral heads are shown in red, green, yellow and brown, respectively. The dashed and solid lines represent the DVHs calculated from the original treatment plan and the reconstructed, respectively.

Data for quasi-3D gamma evaluations with the COMPASS^TM^ system are shown in Table 1. An example of quasi-3D gamma evaluation with 3%/3 mm as well as dose volume histograms (DVHs) of prostate VMAT is shown in Figure 2. All the quasi-3D gamma passing rates were higher than those of the 2D gamma passing rates. The prostate group with both 2%/2 mm and 3%/3 mm and the IMRT group with 3%/3 mm followed the normal distribution of the Shapiro-Wilk test (*p* > 0.05).

For the delivery technique, the average gamma passing rate of the IMRT group with 2%/2 mm was sligntly higher than that of the VMAT group with statistical significance (98.6% ± 0.7% *vs*. 96.6% ± 4.1% with *p* = 0.006). For the treatment site, no statistically significant difference was not observed (all with *p* > 0.05).

The corresponding CLs for each analysis were calculated with the proper confidence coefficient respectively. As in 2D gamma evaluations, institutional tolerance levels for IMRT and prostate plans were higher than those for VMAT and H&N plans, respectively, since the average gamma passing rate of IMRT and prostate plans were higher than those of VMAT and H&N plans.

### Correlation analysis

Since no pairs of both groups followed normal distributions, only Spearman correlation coefficients (*r*) were calculated for correlation analysis between 2D and quasi-3D gamma passing rates.

The calculated correlation coefficients and the corresponding *p* values between 2D and quasi-3D gamma passing rates of IMRT and VMAT are shown in Table 2 with the average value of MU/cGy which represent the degree of modulation. However, the difference in MU/cGy between IMRT and VMAT didn't show the difference in the modulation degree because the technique is different each other. Statistically significant correlation was observed between 2D *vs*. quasi-3D gamma passing rates with 3%/3 mm in the VMAT group (*r* = 0.564 with *p* = 0.012). The others showed no statistically significant correlations.

**Table 2:** Correlation coefficients (*r*) and the corresponding *p* values between 2D and quasi-3D gamma evaluations

	MU/cGy	p*	2%/2 mm		3%/3 mm	
			*r*	*p*	*r*	*p*
**Treatment site**						
Prostate	3.48 ± 0.83	*0.005*	0.137	0.565	0.215	0.363
H&N	5.43 ± 3.29		0.384	0.104	0.448	0.055
**Delivery technique**						
IMRT	6.27 ± 2.51	*< 0.001*	0.096	0.686	0.101	0.672
VMAT	2.63 ± 0.47		0.334	0.162	*0.564*	*0.012*
**Regardless of treatment site and delivery technique**						
			0.239	0.143	*0.372*	*0.020*

*Abbreviations*: MU, monitor unit; H&N, head and neck; p*, *p* value of a statistically significant difference in MU/cGy between prostate and H&N plans; IMRT, intensity-modulated radiation therapy; VMAT, volumetric modulated arc therapy

The correlation coefficients between 2D *vs*. quasi-3D gamma passing rates of prostate and H&N groups are shown in Table 2 with the average value of MU/cGy. The average value of MU/cGy of prostate plans was lower than that of H&N plans, which indicated higher modulation of H&N plans than prostate plans (3.48 MU/cGy *vs*. 5.43 MU/cGy with *p* = 0.005). No statistically significant correlations were observed (always with *p* > 0.05).

The *r* values between 2D *vs*. quasi-3D gamma passing rates regardless of the delivery technique and treatment site with 2%/2 mm and 3%/3 mm were 0.239 (with *p* = 0.143) and 0.372 (with *p* = 0.020), respectively. Statistically significant correlation was observed between 2D *vs*. quasi-3D gamma passing rates with 3%/3 mm, however, the correlation was weak.

## DISCUSSION

In this study, 2D and quasi-3D gamma passing rates of IMRT and VMAT for prostate and H&N cancers were grouped by delivery techniques and treatment sites. The differences in gamma passing rates according to the grouping were statistically analyzed and the CLs for each group were calculated. After that, the correlations between 2D and quasi-3D gamma passing rates were analyzed. Since this study was to investigate the characteristics of 2D and quasi-3D gamma passing rates under the assumption that there was no systematic errors in IMRT and VMAT planning and delivery, we verified delivery accuracy additionally using log files. As shown by the results, the deviations between mechanical parameters of the original plans and those of log files were minimal. In addition, the reconstructed dose distributions using the log files showed no considerable differences in the dose-volumetric parameters compared to those of the original treatment plans. Therefore, the delivery accuracy of both IMRT and VMAT seems acceptable for the treatment of patients [18, 19, 21].

Statistically significant difference was observed between prostate *vs*. H&N plans with 2D gamma evaluation. Since H&N plans were more highly-modulated than did the prostate plans (MU/cGy of prostate and H&N plans = 3.48 *vs*. 5.43), this result was reasonable and similar to those of previous studies [15, 16]. For the quasi-3D gamma passing rates with 2%/2 mm between IMRT and VMAT, we observed statistically significant difference, which was a contradictory result of the previous studies [8, 34]. However, the magnitude of the difference was only 2%, which was minimal.

According to the American Association of Physicist in Medicine (AAPM) Task Group (TG) 119 report, the tolerance level can be acquired by calculating the values of CL [6]. Since the sample size was small and the data were acquired from a single institution, we didn't calculate CLs to acquire tolerance levels for the verification of IMRT and VMAT. Instead, we calculated the CLs for each group to investigate the differences between groups. For 2D absolute gamma evaluations with EBT2 films by measuring an axial dose distribution, CLs with 2%/2 mm and 3%/3 mm were 68.0% and 81.9%, respectively. These values were lower than those recommended by AAPM TG-119 report [6]. Since we additionally verify plan delivery accuracy with log files which showed negligible deviations, we believe that these low gamma passing rates of EBT2 films were caused by uncertainties of film dosimetry not errors of the TPS or beam delivery system. A lot of studies have reported uncertainties of EBT2 film dosimetry due to scanning orientation, non-uniformity of the scanner, film development time, and film uniformity [35, 36]. Moreover, since we performed absolute gamma evaluations with the EBT2 film not the relative gamma evaluation, this might lower the values of gamma passing rates. For quasi-3D absolute gamma evaluations with the COMPASS^TM^ system, much higher values of the CLs than those of 2D gamma evaluations were acquired with both 2%/2 mm and 3%/3 mm, which were 91.5% and 97.5%, respectively. Persoon *et al*. also noted the implementation of 3D gamma evaluations using the same acceptance criteria as those for 2D gamma evaluations would be expected to lead to a higher passing rate [37].

For correlation analysis, no statistically significant correlations were observed between 2D and quasi-3D gamma evaluations except the VMAT group and the group including both IMRT and VMAT with 3%/3 mm. However, the *r* values were not high enough to show strong correlations. As in the previous study by Jin *et al*., no considerable correlation between 2D and quasi-3D gamma evaluations was observed in this study [33]. Since one plane may not represent the other planes’ information within a 3D volumetric dose distribution, it seems reasonable that there was no correlation between 2D and quasi-3D gamma evaluations. High 2D gamma passing rates cannot guarantee the similarity in the volumetric dose distributions between the original plan and delivery. In the same vein, delivered 3D volumetric dose distributions might be similar to those in the original plan despite of poor 2D gamma passing rates in a specific dose plane. Therefore, 2D gamma evaluation, which is a current practice in the clinic, seems not enough to verify plan delivery accuracy of both IMRT and VMAT.

We didn't perform dose-volumetric analysis for each structure with the COMPASS^TM^ system in this study since the aim of this study was to investigate the correlation between gamma passing rates based on 2D information and those based on whole 3D volumetric information. The correlation analyses between quasi-3D gamma passing rates of whole body and clinically-relevant deviations in dose-volumetric parameters were not performed since no clinically relevant differences were observed between the original plan and the reconstructed plan in this study. As a future work, we will perform a study with the clinically unacceptable IMRT and VMAT plans.

No correlations were observed between 2D and quasi-3D gamma passing rates except VMAT. Although VMAT demonstrated weak correlations between 2D and quasi-3D gamma passing rates, it is hard to mention that 2D gamma passing rates could represent quasi-3D gamma passing rates. For more appropriate verifications of both IMRT and VMAT, the verification method based on quasi-3D or 3D information should be performed in the clinic.

## MATERIALS AND METHODS

### Treatment planning and delivery

Ten of each prostate and head and neck (H&N) patients (a total of 20 patients) were retrospectively selected for both IMRT and VMAT planning. Thus, a total of 40 treatment plans were generated for this study. Each IMRT plan had eight coplanar non-opposing isocentric beams (gantry angles of 40°, 60°, 100°, 160°, 200°, 260°, 300° and 320°). Each VMAT plan was generated with two coplanar full arcs. Both IMRT and VMAT plans were generated using a 6 MV photon beam of Clinac iX^TM^ with Millennium 120^TM^ MLC (Varian Medical Systems, Palo Alto, CA, USA). For prostate treatment with both IMRT and VMAT, a primary plan with a prescription dose of 44 Gy with a daily dose of 2 Gy to the primary target volume including both prostate and seminal vesicles was delivered to a patient. After that, a boost plan with a prescription dose of 36 Gy with a daily dose of 2 Gy to the boost target volume that included prostate only was delivered. In this study, only primary plans were analyzed. For H&N treatment with both IMRT and VMAT, a simultaneous integrated boost (SIB) plan with a total of 3 target volumes was delivered to a patient, of which prescription doses were 67.5 Gy (daily dose of 2.25 Gy), 54 Gy (daily dose of 1.8 Gy) and 48 Gy (daily dose of 1.6 Gy). The optimization and dose calculation were done using the Eclipse^TM^ system (version 8.9.17, Varian Medical Systems, Palo Alto, CA, USA). The progressive resolution optimizer (PRO) algorithm was used for the optimization of VMAT while the dose volume optimizer (DVO) algorithm was used for the optimization of IMRT (Varian Medical Systems, Palo Alto, CA, USA) [38, 39]. After optimization, doses for both techniques were calculated using the analytical anisotropic algorithm (AAA, Varian Medical Systems, Palo Alto, CA, USA) with a calculation grid of 2.5 mm [40].

### Acquisition of log files

During beam delivery for the 2D and quasi-3D gamma evaluations for each plan, log files were acquired and analyzed. The actual positions of MLCs during delivery were acquired from the DynaLog files which contain information on the actual MLC positions recorded every 50 ms. In addition, we acquired linac log files which record the actual gantry angles and delivered monitor units (MUs) during VMAT delivery. We reconstructed volumetric dose distributions in patient CT images using the log files. The log files were formatted to correspond to the plan files in DICOM-RT format. Those DICOM-RT files were imported in the TPS and dose distributions were calculated. After that, radiation oncologists examined whether clinically significant deviations exist between the original and the reconstructed dose distributions.

### 2D gamma evaluation

**Figure 3:**
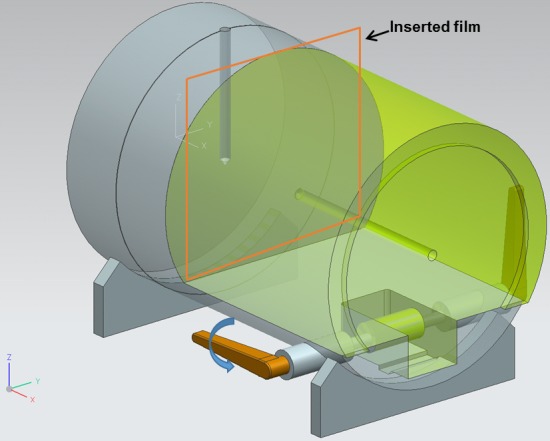
The film was placed between two pieces of a custom-made cylindrical phantom The cylindrical phantom was made of acrylic. The lever was equipped to reduce air gaps between two pieces of the phantom.

The 2D planar dose measurements were performed with radiochromic films (EBT2 films, Ashland Advanced Materials, Covington, KY, USA) to measure dose distributions in an axial plane. The film was placed between two pieces of a custom-made cylindrical phantom, where the isocenter was located at the center of the phantom as shown in Figure 3. The custom-made phantom was designed to eliminate air gaps between the film and the phantom by pulling a lever. Film dosimetry carefully followed the process of self-development provided by manufacturer [41]. Since two batches of films were separately used for the measurements, in order to avoid the interbatch response variation of EBT2 films, which was known to be less than approximately 1%, the films from each batch-numbered packet were used for calibration [42]. The films were scanned 20 hours after irradiation using a flatbed scanner (Epson 10000XL^TM^, Epson Canada Ltd., Toronto, Ontario, Canada) in 48 bit color mode with the practical spatial resolution of 75 dpi. The films were scanned in the landscape orientation for both calibration and measurement. When scanning, the films were located in the central region of the scanner to minimize the effect of non-uniformity of the scanner. To eliminate non-uniformity of the scanner, the background of the scanner was measured and subtracted from the measured dose distributions. The dual channel method of the red and blue correction was applied for calibration [43, 44]. The calculated and measured dose distributions were compared using Verisoft 3.1^TM^ image software (PTW, Freiburg, Germany). The region of interest (ROI) of the film was defined as the size of a 12×10 cm2 rectangle. The 2D gamma evaluation was performed with absolute dose values using the global gamma method. The gamma criteria used for gamma evaluation were 2%/2 mm and 3%/3 mm. The threshold dose which is a parameter to exclude dose points below a selected threshold for the gamma evaluation was set to be 10% of the maximum dose in this study.

### Quasi-3D gamma evaluation

The COMPASS^TM^ system which was configured with measured beam data was used for quasi-3D gamma evaluation. The ion chamber array (MatriXX^TM^, IBA dosimetry, GmbH, Germany) with a build-up material (water-equivalent thickness of 2 cm) was attached to the gantry head orthogonal to the beam direction at the source to surface distance (SSD) of 100 cm. The accuracy of the detector setup was thoroughly evaluated with delivery of three different open fields, equipped with an angular position sensor. After verification of set-up accuracy, actually delivered fluences of IMRT and VMAT plans were measured with an ion chamber array. The sampling interval of the fluence measurements was 250 ms. The measured fluences were applied to the patient CT images, and then the 3D dose distribution at the patient CT images was reconstructed in the COMPASS^TM^ system with the collapsed cone convolution (CCC) algorithm, *i.e*. 3D dose distribution at the patient CT images was calculated with the measured fluences using the the CCC algorithm. The reconstructed 3D dose data were compared to the planned 3D dose data calculated by treatment planning system (TPS) with gamma-index method (*i.e*. quasi-3D gamma evaluation). For the quasi-3D gamma evaluation, the global gamma evaluations with gamma criteria of 2%/2 mm and 3%/3 mm were performed for both IMRT and VMAT. The irradiated patient body was set as ROI of this evaluation. The threshold dose was set to be 10% of the maximum dose same as 2D gamma evaluation.

### Data grouping and statistical analysis

**Figure 4:**
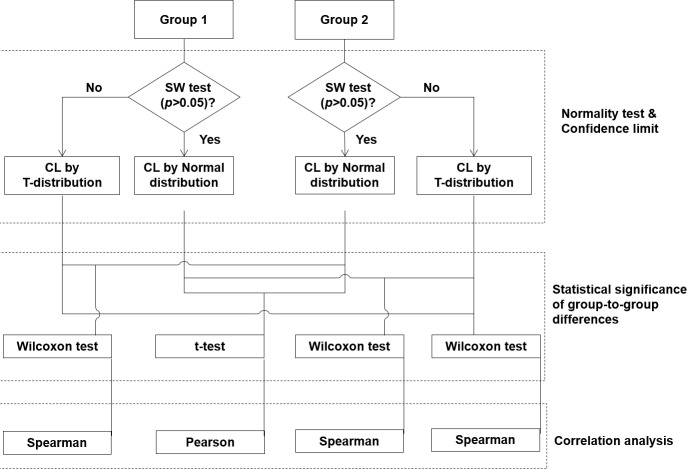
Schematic diagram for grouping data and statistical analysis

The data was grouped by the delivery technique (IMRT *vs*. VMAT) and the treatment site (prostate vs. H&N) for statistical analysis. Thus, the grouping by the delivery technique was done regardless of the treatment site. Similarly, the grouping by the treatment site was done regardless of the delivery technique. The Shapiro-Wilk (SW) test was performed to determine whether a data set of each group is well-modeled by the normal distribution, that is, with the *p* value greater than 0.05 [45]. To assess statistical significance of the differences between two groups, a two sided Student's t-test was used if both groups followed the normal distribution, otherwise, the Wilcoxon rank-sum test was used [46]. The *p* value is equal to or less than 0.05 regarded as statistically significant in this study. For the calculation of CLs, the confidence coefficient of 1.96 was applied to normally distributed groups; otherwise, the confidence coefficient of 2.093 and 2.023 in the Student's t-distribution table of two tails were applied to 20 and 40 samples, respectively, within a 95% confidence level [47]. For correlation analysis, if both groups followed the normal distribution, the Pearson correlation coefficient was calculated; otherwise the Spearman correlation coefficient was calculated to examine the correlations [48]. The correlation analyses were performed between 2D and quasi-3D gamma evaluations with both IMRT and VMAT, with only IMRT and with only VMAT. In addition, correlation analysis between 2D and quasi-3D gamma evaluations were performed with only prostate plans including both IMRT and VMAT (lowly-modulated plans) as well as only H&N plans including both IMRT and VMAT (highly-modulated plans). The schematic diagram of statistical analysis is shown in Figure 4.
